# Ionic Liquids as Unforeseen Assets to Fight Life-Threatening Mycotic Diseases

**DOI:** 10.3389/fmicb.2016.00111

**Published:** 2016-02-08

**Authors:** Diego O. Hartmann, Marija Petkovic, Cristina Silva Pereira

**Affiliations:** Instituto de Tecnologia Química e Biológica António Xavier, Universidade Nova de LisboaOeiras, Portugal

**Keywords:** ionic liquids, filamentous fungi, fungal infections, proteomics, stress response, natural compounds discovery, antifungal drugs

## Abstract

Ionic liquids discovery has celebrated 100 years. They consist solely of ions, one of which is typically organic and asymmetrical. Remarkable physical and chemical properties stirred their use as alternative solvents in many chemical processes. The recent demonstration of their occurrence in nature might boost their interest in biological sciences. In the search of mechanistic understandings of ionic liquids’ ecotoxicological impacts in fungi, we have analyzed the proteome, transcriptome, and metabolome responses to this chemical stress. Data illuminated new hypotheses that altered our research path – exploit ionic liquids as tools for the discovery of pathways and metabolites that may impact fungal development and pathogenicity. As we get closer to solve the primary effects of each ionic liquid family and their specific gene targets, the vision of developing antifungal ionic liquids and/or materials, by taking advantage of elegant progresses in this field, might become a reality. *Task-designed* formulations may improve the performance of conventional antifungal drugs, build functional coatings for reducing allergens production, or aid in the recovery of antifungal plant polymers. The frontier research in this cross-disciplinary field may provide us unforeseen means to address the global concern of mycotic diseases. Pathogenic and opportunistic fungi are responsible for numerous infections, killing annually nearly 1.5 million immunocompromised individuals worldwide, a similar rate to malaria or tuberculosis. This perspective will review our major findings and current hypotheses, contextualizing how they might bring us closer to efficient strategies to prevent and fight mycotic diseases.

These liquid salts have been widely investigated and several hundred are already chemically well characterized. By now, numerous applications of ionic liquids have been proposed, impacting diverse relevant areas, such as Catalysis, Separations, Materials, Sustainable Energies, Biorefineries, Renewable Fuels, and Chemicals, just to name a few. Some remarkable examples include ionic liquids in membranes for gas separation (Tomé et al., 2014), and for the extraction of disease biomarkers and antibodies (Taha et al., 2015). Unfortunately, only a few applications have successfully reached the industrial panorama (Plechkova and Seddon, 2008), e.g., BASIL^TM^ and cellulose reshaping (BASF, Germany), dye sensitizing solar cells (G24i Power, UK) and – certainly one of the most fascinating – the use of mercury grabbing ionic liquids to clean natural gas streams (Abai et al., 2015), commercialized as HycaPure Hg by Clariant (Switzerland).

As a scientific topic, ionic liquids are likely among the most intensely researched ones, especially in the Chemistry, Physics, and Materials disciplines. Over 10000 publications on ionic liquids can be found in the *Web of Knowledge* but little more than 10% intersect with the Life Sciences. So the question that rises from all the above is *why should any environmentalist, biologist or biochemist care about ionic liquids at first?* Perhaps the demonstration that ionic liquids might occur in nature sounds appealing. In a recent study, it was suggested that an ionic liquid is formed during the confrontation of two ant species, *Nylanderia fulva* and *Solenopsis invicta*, as a form of defense mechanism of the former against the venom of the latter ant species (Chen et al., 2014). In line with this idea, it was shown that certain metabolites abundant in plants become liquid when mixed together (Choi et al., 2011). These so-called *natural deep eutectic solvents*, with properties strongly resembling those of ionic liquids, would ensure cellular processes involving water-insoluble compounds. The likelihood of naturally occurring ionic liquids creates a new paradigm – they are not exclusively man-made chemicals – and fosters a new boost of interest in their research.

Ionic liquids and life sciences intertwined for the first time, however, to answer a very fundamental need: understanding ionic liquids’ environmental impact. Our initial motivation, similar to tens of other research laboratories worldwide, was partially due to the fact that, despite being classified as *green* solvents, these organic salts comprise a disparate group of compounds that are not all intrinsically green. Many have been shown to be toxic and recalcitrant to biodegradation. Data collected so far has been compiled in a series of elegant and comprehensive reviews on their environmental impact (Petkovic et al., 2011) and biodegradability (Coleman and Gathergood, 2010). The large majority of early studies on ionic liquids’ toxicity aimed at defining their inhibitory concentrations to very distinct organisms, essentially as to guide chemical research efforts toward more sustainable formulations. These studies have ascertained that different testing models exhibit fairly diverse susceptibilities to ionic liquids, however, often suggesting a similar mechanism of toxicity or cytotoxicity, i.e., plasma membrane permeabilization and oxidative stress (Yu et al., 2009; Petkovic et al., 2011). Unsurprisingly, the chemical nature of the ions rules their specific molecular and/or cellular mode of action. Most toxicity assays, if not all, were based on aqueous systems, in which the composing ions were fully solubilized in water. By accepting this principle, the biological effect of an ionic liquid should consider the individual contributions of its ions. Among the most common cations, the aromatic ones appear to be more toxic than the alicyclic or the quaternary ammonium (Stolte et al., 2007a,b). Nevertheless, the prevailing idea around ionic liquid’s mechanism of toxicity is that, in either of the composing ions, the length of the alkyl chain is directly correlated with lipophilicity and permeabilization of biological membranes, leading to cell death (Zhao et al., 2007). This seems valid only for lipophilic cations, since our most recent data showed that permeabilization by long chain anions (i.e., alkanoates) is hindered by negative charges in the membrane outer surface (Hartmann et al., 2015). These were great news since we have been for long pursuing the use of cholinium alkanoates as novel biocompatible solvents for plant polyesters (Garcia et al., 2010). This idea has been nurturing our research in identifying *task-designed* ionic liquids for the hydrolysis of structural polymers in plant cell walls, i.e., cutin and suberin. Our goal was to preserve the native properties of the polyesters, particularly their function as barriers to microbial pathogens (Ranathunge et al., 2011). In some ways unpredictably, an ionic liquid – cholinium hexanoate – provided us the right means for that. It plays the dual role of solvent and catalyst, promoting the specific cleavage of particular ester bonds of suberin (Ferreira et al., 2014). This ensures the partial preservation of its tridimensional structure, hence the spontaneous formation of films with potentially broad antimicrobial properties (Garcia et al., 2014).

Notwithstanding significant progresses in the field of ionic liquids’ toxicity, our curiosity did not allow us to stop there. We wanted to seek for better mechanistic understandings of how these allegedly man-made chemicals would impact living organisms at a cellular and molecular level. Our front-runner candidates for study organisms were undoubtedly filamentous fungi. Fungi, which are unique and remarkable eukaryotic organisms, act as key colonizers of the soil and ensure major ecosystem functions, including the mitigation of hazardous chemicals (Harms et al., 2011; Varela et al., 2015). Moreover, these organisms are well known as proficient producers of enzymes and metabolites of great biotechnological and pharmacological interest. Several studies revealed that fungal strains commonly found in soil can resist high concentrations of ionic liquids (Petkovic et al., 2009; Singer et al., 2011; Simpson, 2012). In particular our study also demonstrated that all the tested compounds could completely alter the fungal metabolic footprint, i.e., the diversity of diffusible small molecules (Petkovic et al., 2009). These promising and stimulating findings constituted a foundation for our subsequent research efforts.

Looking for a holistic view of the impact of these organic ions in fungal metabolism, we decided to perform a proteomic analysis of model filamentous fungi exposed to ionic liquids (Martins et al., 2013). We specifically selected cholinium chloride and 1-ethyl-3-methylimidazolium chloride, which carry cations currently attracting most academic and industrial interest. These compounds have been previously observed to display very distinct antifungal activities and biodegradability potential (Coleman and Gathergood, 2010; Petkovic et al., 2011). *Aspergillus nidulans* and *Neurospora crassa* – prime model fungal systems for genetic, cellular, and biochemical research – are very dissimilar when accounting for their halo-tolerance (Gunde-Cimerman et al., 2009) and secondary metabolite producing capacity (Khaldi et al., 2010; Inglis et al., 2013). The differential proteome showed that several critical biological processes and pathways were affected by either cation, reflected in the accumulation of numerous stress-responsive proteins and osmolytes, and in the alteration of developmental programs in both fungi. Encouragingly, in this study we observed the accumulation of proteins likely involved in the biosynthesis of non-proteinogenic amino acids in *N. crassa* in the presence of either cation. These rare amino acids are found in secondary metabolites with potent biological activity, e.g., neoefrapeptins and acretocins (Degenkolb et al., 2007; Degenkolb and Bruckner, 2008). Another promising example is the case of *A. nidulans*, which genome has nearly 70 genes coding for multi-domain enzymes likely involved in secondary metabolite biosynthesis (Inglis et al., 2013). Through whole-genome profiling, our recent research efforts revealed that, upon exposure to certain organic ions, this fungus up-regulated a series of secondary metabolism backbone genes (Petkovic and Silva Pereira, 2012). This resulted in a differential metabolic profile that conceals small compounds with biological activities of high pharmacological value (unpublished data). These promising findings open new perspectives on ionic liquids’ potential in the discovery of natural compounds.

The large amount of data that emerge from proteomics or transcriptomics analyses can provide fundamental information on very specific scientific questions. As an excellent example, the resistance of the bacterium *Enterobacter lignolyticus* to 1-ethyl-3-methylimidazolium chloride was in part unraveled using whole genome profiling (Khudyakov et al., 2012). This study arose from the question of how this solvent – able to efficiently dissolve cellulose from plant biomass – could impact biological and fermentation processes. The authors showed that bacteria partially circumvent the toxicity of the cation by increasing membrane transporters and the concentrations of osmolytes. These findings further inspired the design of biofuel cells where ionic liquids are employed for biopolymer dissolution (Ruegg et al., 2014).

As we gather more data from global analyses of the impact of these organic ions, we move deeper into exploring these compounds as tools to solve fundamental questions in fungal biology. Our group rapidly advanced from a rather simplistic view of morphological alterations perceived microscopically (Petkovic et al., 2012) to evaluate, at a gene expression level, membrane and cell wall damage induced by ionic liquids (Hartmann and Silva Pereira, 2013). Fungi can alter the composition of their membranes, regulating its fluidity to overcome adverse environments. The membrane fluidity, which is inversely related to its resistance to permeabilizing compounds, is essentially controlled by the levels of ergosterol and by the balance between saturated and unsaturated fatty acids. The fungal cell wall, on its turn, is responsible for maintaining cell shape, counteracting the turgor pressure and protecting the plasma membrane. Upon damage to the cell wall, fungi respond by activating several genes involved in its biosynthesis, creating conditions that allow them to re-establish its integrity, through the so-called cell wall integrity pathway. This salvage mechanism, better understood in the yeast *Saccharomyces cerevisiae*, remains poorly characterized in filamentous fungi (Fujioka et al., 2007; Valiante et al., 2015). We have demonstrated that some ionic liquids can cause membrane and cell wall damage in *A. nidulans*, most likely activating an alternative cell wall integrity pathway, yet to be characterized (Hartmann and Silva Pereira, 2013). More intriguing is the fact that these organic ions can also activate sphingolipid biosynthesis, leading to the differential accumulation of intermediates, including unknown species (Hartmann and Silva Pereira, 2015). These molecules may participate in the stress response of *A. nidulans*, including the activation of the cell wall integrity pathway. These are noteworthy results, not only for the prospect of unraveling a cross-talk mechanism between the cell wall integrity pathway and sphingolipids biosynthesis, but also because both pathways have for long been considered to be excellent candidate targets for the development of new antifungal agents.

Conventional antifungals, which target, directly or indirectly, the fungal plasma membrane or cell wall, are limited to just a few classes (*viz.* azoles, echinocandins and polyenes) (Odds et al., 2003). New generations of the classical antifungal drugs, as well as non-conventional agents and targets are already available, such as flucytosine and sordarins, which act by inhibiting DNA and protein synthesis, respectively, (Odds et al., 2003). However, clinical development and implementation of new drugs is notoriously long. Hence, the current challenge is to better understand the biology of filamentous fungi, aiming at the discovery of novel targets and the development of new effective drugs and antifungal strategies (Ostrosky-Zeichner et al., 2010; Denning and Bromley, 2015). We hope to make further evident how our cross-disciplinary research will provide means to address these global concerns. As long-term perspective, we seek to deepen our knowledge on fungal biology by exploring organic ions as the right stimuli for deciphering key cellular and molecular processes. We now rely on proteomic tools and, more specifically, phosphoproteomics, to attain deeper insights on the potential elements of the cell wall integrity pathway, as a foundation to solve the puzzling roles of sphingolipids in filamentous fungi.

Although debatable, the intriguing application of ionic liquids in pharmaceuticals development – often mentioned as *third evolution of ionic liquids* – has produced so far notable improvement of drugs solubility, delivery and biological activity through their conversion to a salt form (Hough et al., 2007). This seems a rather interesting prospect, especially when applied to the salt form of the antifungal drug amphotericin B to overcome its low solubility (Petkovic et al., 2015). Nevertheless, the fundamental question we are trying to address is how ionicity impacts the drug primary mode of action. Another path being investigated by us is the use of ionic liquids to reduce the negative impact of pathogens as *Aspergillus fumigatus* by targeting allergen production. There are nearly twenty fully described allergen peptides in this fungus and as many predicted ones (Kurup, 2005; Fedorova et al., 2008). Transcriptomic data suggested that exposure of fungi to certain ionic liquids can strongly reduce the expression of genes coding for putative allergenic peptides (unpublished data). The current challenge is to identify, supported by immunoproteomics, organic ions that strongly interfere with the biosynthesis of allergenic peptides in *A. fumigatus*. This constitutes another elegant example of cell biochemistry manipulation using chemical stimuli and may inspire the use of ionic liquids for developing novel antifungal materials/coatings.

Life-threatening fungal infections present an uprising burden that affects millions of individuals, with more than 2 million invasive fungal infections reported every year worldwide (Brown et al., 2012). Fungi constitute a high risk to immunocompromised individuals of all ages, such as HIV/AIDS, cancer, transplant, and diabetes patients, which represent a significant percentage of the world population. The healthcare costs are enormous, estimated to billions of dollars per year on antifungal drugs only. Mortality rates often exceed 50% even with the current treatment options. This reality is aggravated when considering that the available therapies are sometimes inadequate, as many resistant strains (Anderson, 2005) and emerging fungal pathogens (Fisher et al., 2012) are now being discovered at a regular basis. The identification of new potential risk groups, from asthma sufferers to gastric ulcer patients, further emphasizes the need for efficient antifungal drugs (van Woerden et al., 2013). Our vision is to produce valuable far-reaching insights to advance on the identification and development of novel antifungal strategies to, ultimately, fight fungal pathogenicity.

## Author Contributions

CP conceived and written the first draft; DH and MP contributed in the acquisition, analysis, and interpretation of the data included. All authors revised the manuscript critically for important intellectual content and gave final approval of the version to be submitted.

## Conflict of Interest Statement

The authors declare that the research was conducted in the absence of any commercial or financial relationships that could be construed as a potential conflict of interest.
